# Scholarometer: A Social Framework for Analyzing Impact across Disciplines

**DOI:** 10.1371/journal.pone.0043235

**Published:** 2012-09-12

**Authors:** Jasleen Kaur, Diep Thi Hoang, Xiaoling Sun, Lino Possamai, Mohsen JafariAsbagh, Snehal Patil, Filippo Menczer

**Affiliations:** 1 Center for Complex Networks and Systems Research, School of Informatics & Computing, Indiana University, Bloomington, United States of America; 2 University of Engineering and Technology, Vietnam National University, Hanoi, Vietnam; 3 Department of Computer Science and Technology, Dalian University of Technology, China; 4 Department of Pure and Applied Mathematics, University of Padua, Padua, Italy; The Centre for Research and Technology, Hellas, Greece

## Abstract

The use of quantitative metrics to gauge the impact of scholarly publications, authors, and disciplines is predicated on the availability of reliable usage and annotation data. Citation and download counts are widely available from digital libraries. However, current annotation systems rely on proprietary labels, refer to journals but not articles or authors, and are manually curated. To address these limitations, we propose a social framework based on crowdsourced annotations of scholars, designed to keep up with the rapidly evolving disciplinary and interdisciplinary landscape. We describe a system called Scholarometer, which provides a service to scholars by computing citation-based impact measures. This creates an incentive for users to provide disciplinary annotations of authors, which in turn can be used to compute disciplinary metrics. We first present the system architecture and several heuristics to deal with noisy bibliographic and annotation data. We report on data sharing and interactive visualization services enabled by Scholarometer. Usage statistics, illustrating the data collected and shared through the framework, suggest that the proposed crowdsourcing approach can be successful. Secondly, we illustrate how the disciplinary bibliometric indicators elicited by Scholarometer allow us to implement for the first time a universal impact measure proposed in the literature. Our evaluation suggests that this metric provides an effective means for comparing scholarly impact across disciplinary boundaries.

## Introduction

Many disciplinary communities have sought to address the need to organize, categorize, and retrieve the articles that populate their respective online libraries and repositories. Unfortunately, the great promise of such mechanisms is hindered by the fact that disciplinary categories, as an organizing principle, do not accommodate the trend toward interdisciplinary scholarship and the continual emergence of new disciplines. An initial step towards a solution comes in the form of journal indices, such as those supported by Thomson-Reuters as part of their Journal Citation Reports (JCR) and Web of Science (WoS) commercial products. Systems like the Web of Science, and similar discipline classifications such as MeSH for life sciences, PACS for physics, and ACM CCS for computing, are based on a *top-down* approach in which the ontology is maintained by dedicated curators. However, as disciplines evolve through novel discoveries and interdisciplinary collaborations, semantic predicates associated with these ontologies may become increasingly vague and less informative and will fail to identify the interdisciplinary work occurring at the granularity level of articles and the new areas that emerge at the disciplinary boundaries.

The “Web Science” paradigm suggests an alternative approach. Rather than attempting to match new scientific production to predefined categories, it would be useful to facilitate semantic evolution by empowering scholars to annotate each other's work. This *bottom-up* approach has already been adopted in popular systems such as Bibsonomy.org [1], Mendeley [2], and many others [3], [4]. There are at least three benefits to such a *crowdsourcing* model: (i) dynamic classification that scales with the growth of the number of authors, articles, and specializations [5]; (ii) flexibility to capture emergent interdisciplinary fields compared to hierarchical taxonomy [6]; and (iii) emergence of structure and consensus from the shared vocabularies of interdisciplinary collaborators [7], [8].

Disciplinary boundaries create similar hurdles for measuring scholarly impact, although these hurdles are relegated more to standards and practices. For example, the fields of history and physics have very different publishing patterns and standards of collaboration. A historian may work for years to publish a solitary work while an experimental physicist may co-author numerous articles during the same time period. How do we compare scholars across fields?

Radicchi *et al.* [9] found that citations follow a universal distribution across disciplines when certain discipline-specific statistical quantities are taken into account. Thus, they reasoned, one could construct a universal impact measure to compare authors across disciplines. However, such discipline specific statistics are not available from established bibliometric sources. The crowdsourcing model described above has the added advantage that when combined with citation information about the authors, it can enable the collection of statistical data necessary for the computation of cross-disciplinary impact metrics.

What we envisage is crowdsourcing the knowledge of community members in a scenario similar to those explored in citizen science [10] and games with a purpose [11]. Users would provide disciplinary annotations in exchange for access to citation data obtained from querying bibliographic services (e.g., Google Scholar, CiteSeer, Scopus, and Web of Science). The combined annotation and citation data could then be freely shared with the public. In practice, the idea is to provide a social client interface to an extant Web source of scholarly data, allowing users to perform academic impact analysis based on author queries. This means the data will originate from two general sources: (i) citation data will be collected from public and private sources online, and (ii) users will annotate authors with discipline tags.

Scholarometer is a social tool for scholarly services developed at Indiana University, with the dual aim of exploring the crowdsourcing approach for disciplinary annotations and cross-disciplinary impact metrics [12]. These two aims are closely related and mutually reinforcing. The annotations enable the collection of discipline specific statistics, and therefore the computation of universal impact metrics. In turn, the service provided to users by computing these metrics works as an incentive for the users to provide the annotations.

The goal of this paper is to detail the design and implementation of the Scholarometer tool. We present visualization and data exchange services that are fueled by the data crowdsourced through Scholarometer. We also outline the computation of both disciplinary and universal rankings of authors enabled by this data. In particular, we make the following contributions:

- We present the architecture, user interface, and data model used in the design and implementation of the Scholarometer system. We discuss several heuristics employed to deal with the noisy nature of both bibliographic data and user-supplied annotations (Materials and Methods section).
- As an illustration of potential applications of crowdsourced scholarly data, we report on data sharing and interactive visualization services. These applications suggest that the crowdsourcing framework yields a meaningful classification scheme for authors and their disciplinary interactions (Data Sharing and Visualization section).
- By leveraging socially collected discipline statistics, we implement the so-called “universal 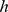-index” proposed by Radicchi *et al.* [9]. This is the first implementation that makes the metric publicly available. We show that user-provided tags provide stable disciplinary coverage, and that the universal 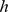-index can be a reliable indicator for comparing the scholarly impact of individual authors across different disciplines (Results section).

### Background

Tools exist for both citation analysis (e.g., Publish or Perish [13]) and social management of bibliographic records (e.g., Mendeley [2]). To our knowledge, Scholarometer is the first system that attempts to couple these two functions with the goal of achieving a synergy between disciplinary annotations and universal impact metrics [12].

The extraction of bibliographic information from online repositories is not new. Bibliographic management tools such as BibDesk offer robust search of online resources and digital libraries like PubMed [14]; users can import objects into Connotea using Digital Object Identifiers (DOI) [3]; and Zotero can spider through and collect bibliographic information from webpages [15]. These and many other bibliographic management tools are compared in Wikipedia [16].

Social tagging of scholarly work is not a new idea, either [3], [4], [17]. In the folksonomies that result from these social tagging systems, tags are assigned to papers. In Scholarometer, users tag authors instead. This makes it possible to collect disciplinary annotations in a more convenient way for the users. Further, we emphasize the use of scholarly disciplines in the annotations, as discussed in the sectionUser Iterface.

We have chosen to use Google Scholar as the citation database for our research. Web of Science, Scopus, and Microsoft Academic Search are possible alternatives [18], but Google Scholar has the advantage of being free and comprehensive, claiming to cover articles, theses, books, abstracts, and other scholarly literature from all areas of research [19]. Our preliminary analysis suggests that Google Scholar has a higher coverage than Microsoft Academic Search [20].

An important goal of the proposed annotation crowdsourcing platform is to enable the computation of scholarly impact. Bibliometrics is the use of statistical methods to analyze scholarly data and identify patterns of authorship, publication, and use. Constitutive of bibliometrics is citation analysis, used to measure the impact or influence of authors and papers in a particular field. There is a plenitude of citation measures. Some (e.g., Hirsch's 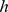-index [21]) balance productivity and impact by trading off between number of publications and number of citations; others seek to apportion the proper weight for highly cited publications (e.g., Egghe's 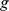-index [22]) or apportion citations fairly for papers with multiple authors (e.g., Schreiber's 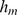-index [23]); and still others (e.g., Radicchi *et al.*' s universal 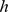-index [9]) attempt to quantitatively compare the impact of authors across disciplines. Scholarometer implements multiple citation measures including 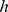-index, 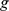-index, 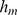-index and universal 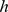-index. We compare the 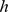-index with the universal 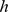-index, as discussed in the section Impact Analysis and Universality.

Scholarometer's crowdsourcing method, in which annotation data is generated by users in exchange for a service, is grounded in prior work as well. Amazon's Mechanical Turk [24], Wikipedia [25], and GalaxyZoo [10] are popular examples of crowdsourcing. This technology coordinates the application of human intelligence as a stopgap in problems that computers are unable to solve. For example, people may prove more capable at describing or judging certain objects such as a picture or piece of music. The ESP ‘game with a purpose’ [11] is another forerunner of Scholarometer, in which users generate useful annotation data in exchange for entertainment [26], [27].

## Materials and Methods

In this section we outline the main features of the Scholarometer system, available at scholarometer.indiana.edu.

### Architecture

Any citation analysis tool can only be as good as its data source. As mentioned earlier, Scholarometer uses Google Scholar as a data source, which provides freely accessible publication and citation data to users without requiring a subscription. Google Scholar provides excellent coverage, in many cases better than the Web of Science — especially in disciplines such as computer science, which is dominated by conference proceedings, and some social sciences, dominated by books. Nevertheless, Google Scholar is based on automatic crawling, parsing, and indexing algorithms, and therefore its data is subject to noise, errors, and incomplete or outdated citation information. The data collected from Google Scholar comprises the number of papers by an author along with their citation counts and publication years. Alternative sources, such as Microsoft Academic Search (academic.research.microsoft.com) or CiteSeer (citeseerx.ist.psu.edu), can provide the same data for the queried author. Therefore, the system architecture and design that we describe below are independent of the data source.

Due to the lack of an API to access Google Scholar data, a server-based implementation would violate Google Scholar's policy about crawling result pages, extracting data (by scraping/parsing) and making such data available outside of the Google Scholar service. Indeed, server-based applications that sit between the user and Google Scholar are often disabled, as Google Scholar restricts the number of requests coming from a particular IP address. Workarounds such as configurable proxies are not desirable solutions as they also appear to violate policy. We further excluded Ajax technology due to the same origin policy for JavaScript, and the gadget approach because it would render the tool dependent on a particular data source. We turned to a client-based approach, but ruled out a stand-alone application (such as Publish or Perish) for portability reasons. These design considerations led us to a browser extension approach, which is platform and system independent and, to the best of our knowledge, in compliance with Google's terms of service.

In keeping with the above considerations, Scholarometer is implemented as a smart browser extension, through which the user queries the source, annotates the results, and shares with the Scholarometer community only annotation metadata from the users and public citation data. We emphasize that Scholarometer does not store a copy of a subset of the Google Scholar database. In particular, the records returned to the users from Google Scholar are not stored. The data that our system collects from the users comprises of publication year, number of citations, and number of authors for each article. This information is open for the users to share with the community.

**Figure 1:**
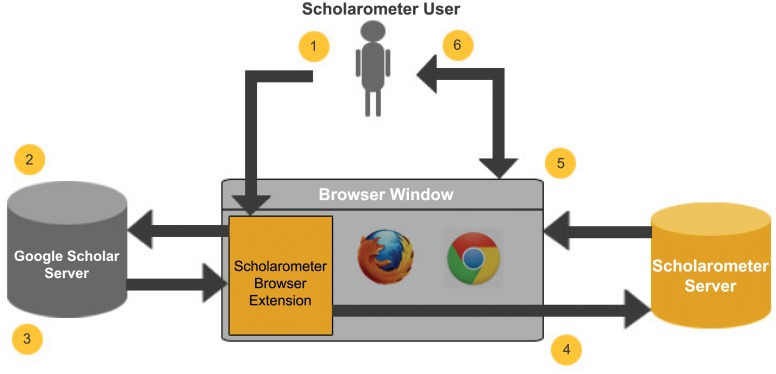
The Scholarometer workflow.

The architecture and workflow of Scholarometer is illustrated in Figure 1. There are six steps: (1) The user enters a query and discipline tags for an author into a search form provided by the browser extension. (2) The browser extension forwards the query to Google Scholar. (3) Google Scholar returns the query results to the browser. (4) The browser extension then forwards the results to the Scholarometer server. This parses the results to extract citation and other metadata, which is then inserted into the database, along with annotation metadata. (5) The Scholarometer server sends to the client browser the bibliographic records and impact measures for the queried author(s). (6) Finally, the client browser renders the data in an interactive way. The user views results in a new browser tab and can perform advanced actions such as sorting, filtering, deleting, and merging records.

### User Interface

**Figure 2:**
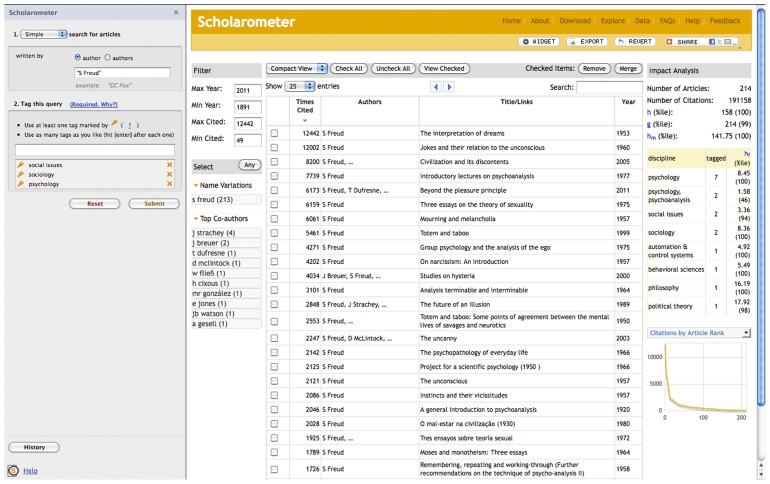
Illustration of the Scholarometer interface.

The Scholarometer tool has two interfaces for communicating with users: one in the browser extension for entering queries and tags, the other in the main browser window for presenting and manipulating bibliographic data and citation analysis results. The browser extension is available in two versions: one for the Firefox browser hosted at the Mozilla Firefox Add-ons site, and one for Chrome browser hosted at the Google Chrome Web store (scholarometer.indiana.edu/download.html). The Firefox interface is illustrated in Figure 2.

The query interface in the browser extension is designed to identify one or more authors and retrieve their articles. The default interface hides many advanced features and simplifies the common case of a single author uniquely identified by name. Advanced interfaces are available with explicit Boolean operators for multiple authors or ambiguous names, with controls for filtering subject areas and languages, and with additional keyword fields.

Tagging a queried author with disciplinary annotations is a key requirement of the extension interface. We considered two possibilities for the set of usable tags. One is the use of a predefined, controlled vocabulary. This closed approach has the advantage of producing “clean” labels, but the limitation of disallowing the bottom-up, user-driven tracking of new and emerging disciplines, which is a crucial goal of our project. At the other extreme, the open approach of free tagging addresses the latter goal but opens the door to all kinds of noise, from misspelled keywords to the use of non-disciplinary labels that can be useful to a particular individual but not necessarily to the community — think of tags such as “ToRead,” “MyOwn,” “UK,” and so on. We therefore aimed for a compromise solution in our design. The user must enter at least *one* annotation from a controlled hierarchical ontology of disciplines, and can enter any free tags without additional constraints. The predefined labels are the set of subject categories extracted from the three major Thomson-Reuters citation indices (*Science Expanded*, *Social Science*, and *Arts & Humanities*). This way each queried author is associated in the Scholarometer database with at least one established subject category and one of the three high-level classes. In addition, we provide an autocomplete feature to make it easy for users to enter discipline tags and reuse tags from other users, thus decreasing the frequency of misspellings.

The interface in the main browser window is designed to facilitate the manipulation and cleaning of the results, to visualize how the impact measures are calculated, and to expose annotations from other users for the same author(s). The output screen is divided into three panels:

1. A filter panel with two modules. One module is for pruning the set of articles based on the publication year or the number of citations. The second module is for limiting the set of articles to selected name variations or co-authors.
2. The list of articles, with utilities for live searching and for alternating between a simplified and an extended view, as well as links to external resources. This panel also has remove and merge utilities to correct two common sources of noise in Google Scholar results: articles written by homonymous authors and different versions of the same paper.
3. A citation analysis panel reporting impact measures. As discussed in the section sec:background, many impact measures have been proposed, and it is infeasible to implement them all. Since a single measure can only capture some aspect of scientific evaluation, a good citation analysis tool should incorporate a set of measures that capture different features, such as highly cited publications, co-authorship, and different citation practices. To this end we have implemented Hirsch's 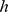-index [21], Egghe's 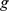-index [22], Schreiber's 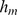-index [28], and Radicchi *et al.*' s index that we call 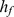 [9]. Note that this is the first implementation of the universal 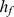-index available to the public, which is enabled by the joint availability of annotation and citation data, as explained in detail in the section hf. The citation analysis panel displays 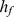 values for each discipline tag of an author, along with percentiles. Finally, the panel shows two plots illustrating the citation distribution and publications per year. All the data in the citation analysis panel is dynamically generated and updated in response to any filter, merge or delete action performed in the other panels.

To provide additional incentives for users to submit more queries, thus contributing more annotation data, we offer the functionality of exporting bibliographic records from the main browser window. Publication data can be exported individually or in bulk into formats commonly used by reference management tools and scholarly data sharing services. At present, Scholarometer supports the following formats: (BIB), RefMan (RIS), EndNote (ENW), comma-separated values (CSV), tab-separated values (XLS), and BibJSON [29]. Note that the publication data is generated dynamically at query time and not stored on our server, except for a temporary cache. Since the data provider (Google Scholar in our case) makes the bibliographic records freely accessible to end users and not our service, it is up to the users to save this information for local use or for sharing more broadly. Data that can be exported also includes citation counts and disciplinary annotations. This helps users who plan to share the data via scholarly tagging systems, such as BibSonomy, and facilitates the propagation of socially-vetted tags.

### Query Management Heuristics

**Figure 3:**
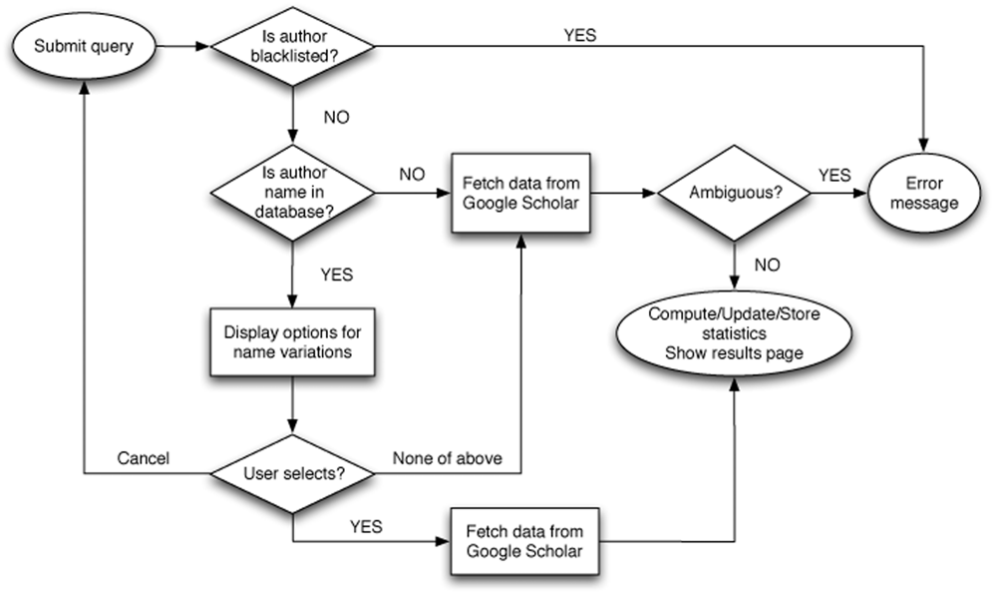
A flow-chart illustrating how queries are handled by the Scholarometer query manager, employing heuristics to deal with problematic and existing author names.

The data that we collect comes from users, so it is naturally noisy and subject to various issues. We propose several heuristics to deal with these sources of noise. Figure 3 illustrates how these heuristics are integrated into Scholarometer's query manager.

We employ a blacklist to prevent spammers from polluting our database. An example is the fictitious author “Ike Antkare,” fabricated to highlight the vulnerability of online sources of citation data [30]. When a query matches a name from the blacklist, the system generates an error message. Fraudulent names are manually added to the blacklist by system administrators.

A critical challenge for bibliometric services is that author names are often ambiguous. Ambiguous names lead to biased impact metrics. The problem is amplified when names are collected from heterogeneous sources, including crowdsourced annotations. This is the case in the Scholarometer system, which cross-correlates author names in user queries with those retrieved from bibliographic data. A component of the Scholarometer system therefore attempts to detect an ambiguous name at query time. When an author name is deemed ambiguous, the user is prompted to refine the query. This design aims at decreasing noise in the database and limiting inaccurate impact analysis.

Our first attempt to deal with ambiguous author names deployed a simple heuristic rule based on citation counts associated with name variations [12]. With the increasing popularity of the tool, the number of authors in the Scholarometer system has grown significantly, revealing that many ambiguous names were undetected. We thus introduced a supervised learning approach to detect ambiguous names at query time, based on a combination of features. We extended the original heuristics by exploring a feature that measures the consistency among the topics associated with publication metadata, with the help of crowdsourced discipline annotations. The accuracy is about 75% [31].

Work on the ambiguous name detection problem is ongoing. We are currently exploring the incorporation of additional features into the classifier. One new class of features under study is based on metadata consistency. We developed a two-step method to capture the consistency between coauthor, title and venue metadata across publications. Authors are likely to collaborate with a certain group of authors, write papers with related titles, and publish papers in similar journals or conferences. The metadata associated with these publications by the same author should be consistent. Another new feature is the consistency between topics associated with publication metadata and discipline annotations crowdsourced from the users. By combining all these features, the accuracy reaches almost 80% [20].

Since there is no established way to uniquely identify authors (the ORCID initiative is under development [32]), we use a signature of the query as an author identifier. Keywords used in queries contribute to the generation of unique identifiers. To reduce duplicate author records, the system uses the following rules when a query is submitted (see Figure 3):

1. If the author name is already present in the database, the system prompts the user to make a selection from a list of names provided along with citation metadata.
2. If the user chooses someone from the list, the system updates the information for the author rather than creating a new record.
3. If the user does not choose a name from the list, but an author generated from an identical query is present in the database, the user is prompted to use additional keywords to disambiguate the query.
4. If the user does not choose a name from the list, and an identical author is not present in the database, a new record for the author is created.

A second issue is the arbitrary nature of uncontrolled discipline annotations. As mentioned earlier, free tags can be noisy, ambiguous, or duplicated. We employ manual and automatic techniques to deal with noisy annotations. We found different types of noise in our tag collection. First, some users misunderstand the tagging request, and utilize author names instead of discipline names as tags. Second, misspelled disciplines names are common, resulting in a duplication of existing tags. Third, some users adopt acronyms without checking if an extended version of the discipline name already exists (e.g., “hci” vs. “human computer interaction”). Finally, people may abuse the tool, using non-sensical or random tags, e.g., the first discipline starting with the letter ‘a.’

Some of these issues can be dealt with automatically by (i) checking if a tag corresponds to an author name present in the database, and (ii) ordering all tags in lexicographical order and calculating the edit distance within a window of neighboring tags. We employ the Damerau-Levenshtein (DL) distance [33] to this end. All the pairs of tags whose DL distance is less than a fixed threshold (currently 2) are flagged. The tag with lower use in each pair is merged into the one with higher use, with manual supervision. A tool developed for internal use allows system administrators to perform these tasks, as well as to remove junk tags and manually merge tags where appropriate, e.g., a user generated tag such as “AI” that duplicates the predefined “computer science, artificial intelligence” discipline.

**Figure 4:**
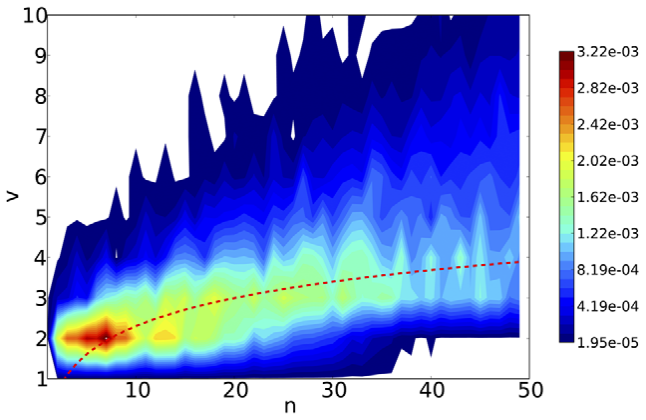
Entropy contours of a model in which tags are drawn from the overall distribution of votes. When a tag is selected it receives a vote, bringing its total number of votes to 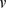. There are 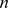 tags with at least one vote. We plot the area in which the average change in entropy 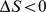. The colors represent the magnitude of the decrease in entropy, 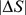. Our heuristic threshold 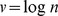, also plotted, tries to capture the number of votes that results in the largest decrease in entropy, making a tag reliable.

Finally, we need a way to estimate the reliability of crowdsourced discipline tags. We view each query as a vote for the discipline tags of the queried author. For example, a query that tags Einstein with “physics” and “philosophy” generates a vote for (Einstein, “physics”) and a vote for (Einstein, “philosophy”). The number of votes together with the number of tags can be used to determine heuristically which tags are *reliable* for each author. The intuition is that the more tags an author has, the greater the possible confusion, and proportionally the greater the number of votes necessary for a tag signal to rise above the noise. To derive a heuristic according to this intuition, we simulated a model in which votes are assigned to tags for an author by randomly drawing from the overall distribution of votes. Suppose that an author has 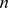 tags that have received 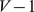 votes collectively, and a new vote is assigned to tag 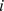 resulting in 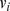 votes for 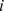 out of a total of 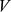 votes. Note that 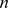 increases if 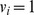. We can measure the change in entropy 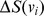 corresponding to this new vote, where 
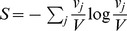
. According to our intuition, tag 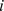 is reliable if 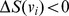, i.e., the confusion as measured by the entropy decreases as a result of the number of votes accrued by 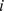. We simulated this model 1000 times, using a total of 1000 tags and stopping each run when 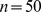. We then averaged the change in entropy 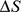 corresponding to the combination of 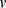 and 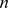 values for each new vote. In Figure 4 we show that according to this model, the number of votes 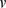 necessary to make a tag reliable grows slowly with the number of tags 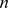. We chose to adopt a heuristic threshold: a tag is deemed reliable for an author with 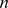 tags if it has 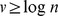 votes.

### Data Sharing and Visualization

Scholarometer provides several ways to share the crowdsourced data with the research community, and to explore the data through interactive visualizations.

The API (scholarometer.indiana.edu/data.html) makes the data collected by Scholarometer available. It also makes it easy to integrate citation-based impact analysis data and annotations into other applications. It exposes information about authors, disciplines, and relationships among authors and among disciplines.

The Widget provides an easy and customizable way to embed a dynamically updated citation analysis report into any website. The results screen in the main browser window includes a special “widget” button (see Figure 2) leading to code and instructions to embed the citation analysis report. The widget feature can also be accessed from the home page (scholarometer.indiana.edu/cgi-bin/widget.cgi).

**Figure 5:**
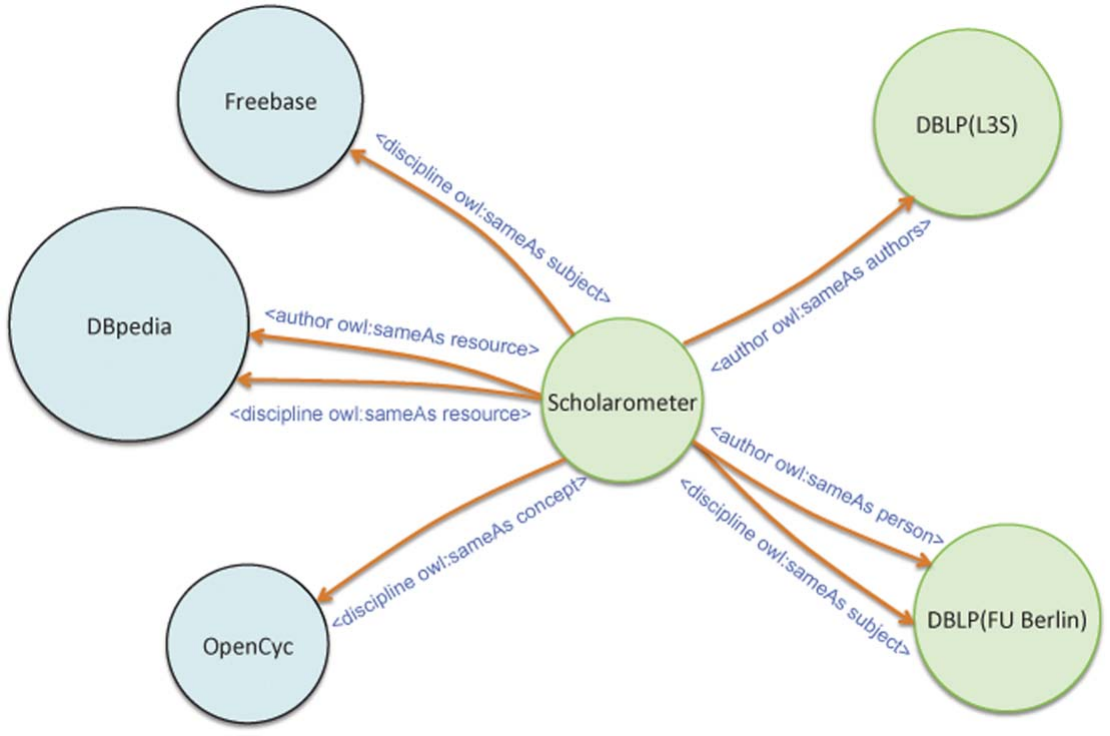
Connections between Scholarometer and other Linked Open Data sources. Links are labeled with the correspondence relationships between resources. This diagram is a portion of the cloud diagram by Richard Cyganiak and Anja Jentzsch (lod-cloud.net). As in the original cloud diagram, the color of a node represents the theme of the data set and its size reflects the number of triples.

Scholarometer also publishes crowdsourced data according to the basic principles of “Linked Data” [34] (scholarometer.indiana.edu/data.html). The aim is to make information about authors and disciplines based on citation analysis available on the Semantic Web. Linked Data is a style of publishing and interlinking structured data on the Semantic Web. Data is described and linked using a language called RDF (Resource Description Framework). Users can use generic RDF browsers (e.g., Tabulator, Disco, OpenLink Browser), RDF crawlers (e.g., SWSE, Swoogle), and query agents (e.g., SemWeb Client Library, SWIC) to explore the data. We assign URIs (Uniform Resource Identifiers) to authors and disciplines and implement an HTTP mechanism called “content negotiation” to provide an HTML representation in addition to the RDF representation of a resource. Linked Data encourages interlinks between different data sources, which enable Semantic Web browsers and crawlers to navigate between them. As illustrated in Figure 5, Scholarometer RDF links primarily point to DBpedia, DBLP, Freebase and Opencyc data sets using the owl:sameAs property, which indicates that two URIs refer to the same thing.

**Figure 6:**
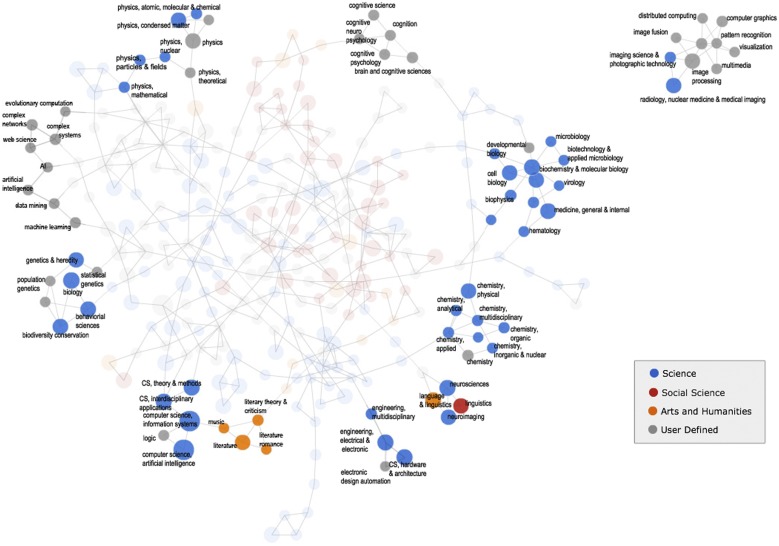
Interactive visualization of discipline network, available on the Scholarometer website (scholarometer.indiana.edu/explore.html).

One way to explore the quality of the annotations obtained through the crowdsourcing approach employed by the Scholarometer system is to map the interdisciplinary collaborations implicit in the tags. Since an author can be tagged with multiple disciplines, we can interpret such an annotation as an indicator of a link between these disciplines. For example, if many users tag many authors with both “mathematics” and “economics” tags, we can infer that these disciplines are strongly related, even though they belong to different branches of the JCR — science and social sciences, respectively. Figure 6 presents a network that visualizes the relationships between the tags in Scholarometer based on the number of authors annotated with each tag. The nodes in the network represent disciplines. Each node's area is proportional to the number of authors in the corresponding discipline, i.e., the total number of authors tagged with that discipline. Nodes corresponding to JCR categories are colored based on the ISI citation indices: blue for science, red for social sciences, and orange for arts and humanities. User-defined disciplines are represented by gray nodes. We see a predominance of scholarly data in the sciences based on current Scholarometer usage. The presence of large gray nodes underlines the limits of the JCR classification. Edges represent interdisciplinary collaborations, as induced by author annotations. We represent each discipline as a vector of authors, where each coordinate is the number of votes assigning the corresponding author to a discipline. An edge connecting two disciplines has a weight equal to the cosine similarity between the two vectors. The more common authors who are tagged with both disciplines, the stronger the weight. The interactive visualization also displays the top authors in a discipline and their impact metrics when a user hovers over the node. The layout of the network is obtained by Fruchterman and Reingold's force-directed algorithm [35], so that related disciplines are more likely to be near each other. The plausible map of science that results from our annotations, as illustrated by the highlighted areas in Figure 6, suggests that the crowdsourcing framework yields a meaningful classification scheme for authors and their disciplinary interactions.

**Figure 7:**
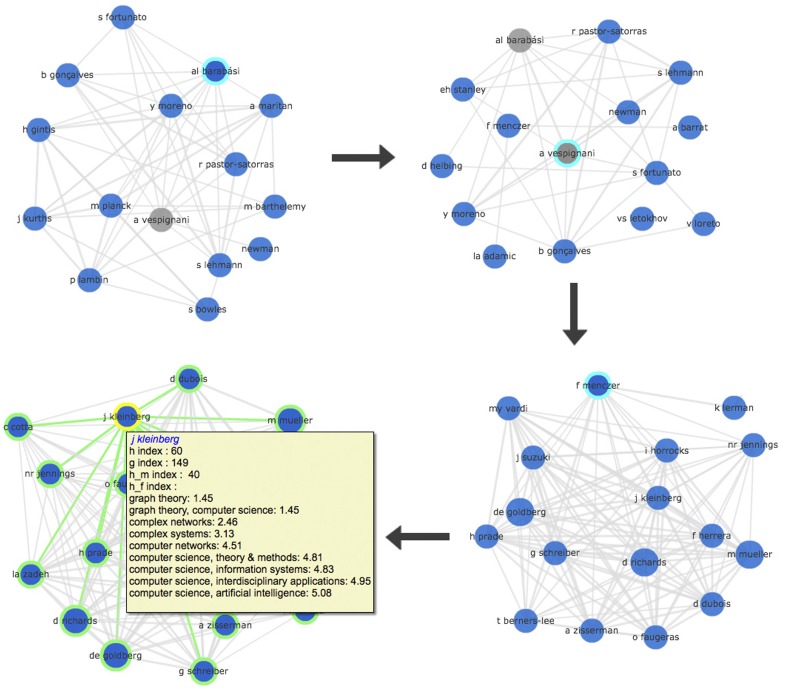
Networks of similar authors, available on the Scholarometer website (scholarometer.indiana.edu/explore.html). In this example scenario, the user is looking for potential members of an interdisciplinary panel on complex networks. Starting from a known physicist (“A L Barabási”) and navigating through “A Vespignani” and “F Menczer,” the user identifies “J Klienberg,” a computer scientist who studies networks.

Along with interactive discipline network, Scholarometer also provides interactive visualizations of author networks. Starting from an author submitted in a query, the author network displays similar authors. An author is represented as a vector of discipline tags, weighted by votes. Author nodes are connected by an edge weighed by the cosine similarity between the corresponding vectors. Authors are therefore deemed similar if they are tagged similarly. These visualizations can help identify potential referees, members of program committees and grant panels, collaborators, and so on. Such a scenario is illustrated in Figure 7. Author nodes are colored based on their predominant reliable tag. The tooltip of a node in the network displays the corresponding author's impact metrics.

## Results

### Data Analysis

**Figure 8:**
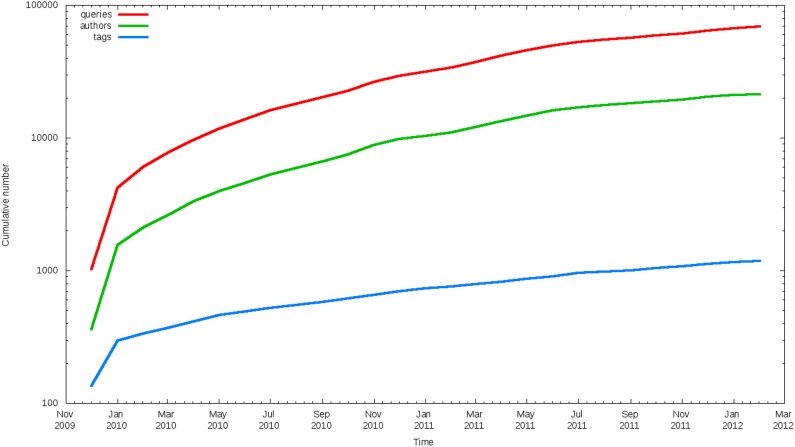
Temporal growth in numbers of authors, disciplines, and queries received by the Scholarometer system.

**Figure 9:**
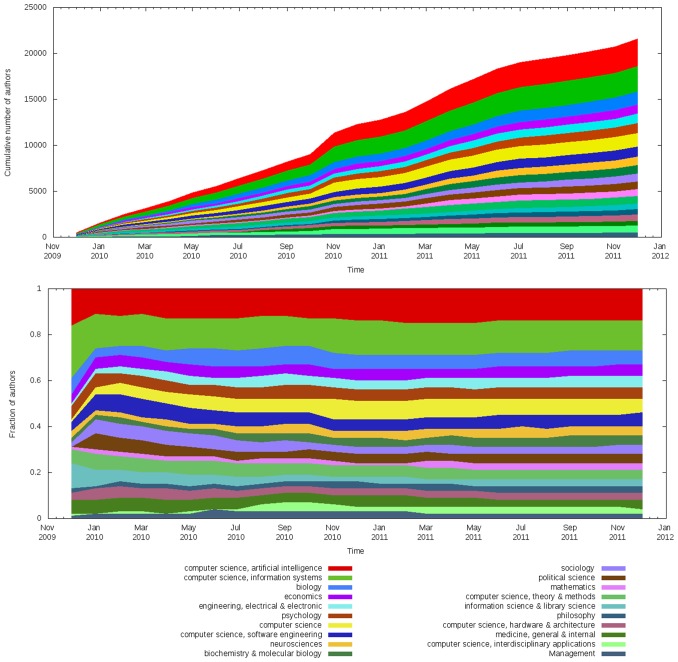
Top: Number of authors tagged with 20 most common disciplines over time. Note that the sets of authors in these disciplines may overlap, as authors are often tagged with multiple disciplines. Therefore the total number of unique authors in these 20 disciplines is actually lower than shown here. Bottom: Relative size of top 20 disciplines based on the number of tagged authors.

The Scholarometer system was first released in November 2009. At the time of this writing the Scholarometer database has collected information about 1.9 million articles by 26 thousand authors in 1,200 disciplines. There are about 90 thousand annotations, or tag-author pairs. Once we apply the heuristics described in the section heuristics, we reduce these numbers to about 1.4 million articles by about 21 thousand reliable authors with about 34 thousand reliable annotations into about 900 reliable disciplines. Naturally this folksonomy grows and evolves daily as Scholarometer handles new queries. The growth in the numbers of discipline tags, authors, and queries is charted in Figure 8, illustrating an initial phase of exponential growth followed by a steady linear regime. Figure 9 (top) displays the dynamics of the top 20 discipline tags, based on number of authors. To better illustrate the proportions of authors in the various disciplines, the ratios are plotted in Figure 9 (bottom). We observe that the Scholarometer database was initially dominated by computing-related disciplines, due to the publicity received by the tool in the computer and information science community. Over time, the collection has become more uniform and the coverage of various disciplines has grown.

Various statistics for authors and disciplines are available on the Scholarometer website (scholarometer.indiana.edu/explore.html). The annotation data enables us to derive rankings for authors — both universal and disciplinary — based on impact metrics. Table 1 shows the universal rankings of top authors by 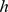, 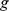, 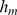, and 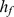 respectively. We can see that compared to the 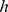-index, the 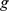-index favors authors such as D.R. Cox and A. Shleifer, with books that have received very high numbers (thousands) of citations. The 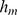-index favors authors with many top publications that are single-authored; M. Friedman and physics nobel prize winner S. Weinberg are good examples. The universal metric 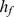 (discussed next in section hf) brings to the top some authors whose citations are not as numerous in absolute terms, but who are leaders in their respective fields — nobel prize winner in economics P. Krugman and C.R. Sunstein from law are good examples.

**Table 1:** Top authors according to various impact measures (based on values as of January 2012).

	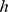	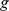	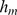	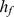
1	JN Ihle	DR Cox	S Freud	S Freud
2	WC Willett	GM Whitesides	M Friedman	N Chomsky
3	MJ Stampfer	JN Ihle	P Bourdieu	S Kumar
4	M Friedman	W Zhang	SH Snyder	W Zhang
5	W Zhang	S Freud	E Witten	CR Sunstein
6	SH Snyder	LA Zadeh	JE Stiglitz	R Langer
7	Y Sun	A Shleifer	S Weinberg	E Witten
8	S Freud	P Bourdieu	N Chomsky	P Krugman
9	B Vogelstein	N Chomsky	HA Simon	P Bourdieu
10	S Kumar	T Maniatis	RA Posner	JL Goldstein

Table 2 shows an example ranking of top authors in a particular discipline (“computer science, artificial intelligence”) by the same impact measures. Once again we observe that the 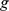-index ranks higher authors of books and other very highly cited publications, such as D.E. Knuth and S. Haykin. D. Dubois and L.A. Zadeh have many top cited single-authored articles and as a result are highly ranked by 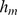. Finally, in the ranking by 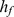 we see that some authors with high 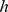 are replaced by other well-known researchers in artificial intelligence, such as J. Kleinberg, O. Faugeras, and A. Halevy. The 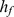 metric is discussed in more detail next.

**Table 2:** Top authors tagged with “computer science, artificial intelligence” according to various impact measures (as of January 2012).

	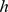	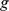	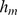	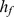
1	DE Goldberg	LA Zadeh	LA Zadeh	LA Zadeh
2	S Thrun	DE Goldberg	DE Knuth	NR Jennings
3	NR Jennings	AL Barabasi	DE Goldberg	AL Barabasi
4	D Dubois	DE Knuth	D Dubois	A Zisserman
5	LA Zadeh	S Haykin	S Thrun	I Horrocks
6	A Zisserman	NR Jennings	NR Jennings	J Peters
7	AL Barabasi	G Salton	H Prade	J Kleinberg
8	H Prade	M Dorigo	JY Halpern	O Faugeras
9	DE Knuth	A Zisserman	A Zisserman	S Thrun
10	I Horrocks	D Dubois	MY Vardi	A Halevy

### Impact Analysis and Universality

The universal 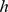-index, which we refer to as 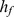, was proposed by Radicchi *et al.* [9]. For each discipline tag and year, we maintain statistics about the average number 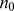 of papers written by authors in that discipline and in that year, and about the average number 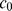 of citations to papers written in that discipline and in that year. When we receive a query about an author in a certain discipline, we update these statistics. Following Radicchi *et al.*, we rescale the number of citations 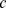 of each paper. This is done by dividing 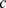 by 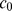 (for the discipline of the author and the year of the paper). Papers are then ranked by the rescaled number of citations 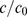. Similarly, we divide the resulting rank of each paper by 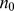 (again for the given discipline and year). The universal 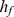 value for the author is defined as the maximum rescaled rank 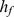 such that each of the top 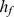 articles have at least 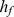 rescaled citations each.

Note that an author tagged with several disciplines will have multiple 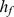 values, one per discipline. Since different disciplines have different citation patterns, an author should only pay attention to 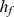 values in disciplines that s/he knows to be appropriate.

Since the discipline/year statistics depend on the annotations we collect from queries, they are subject to noise and may take a while to converge. Once the statistics are reliable, one should in theory be able to compare the impact of authors in different disciplines. Given the dependence of 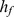 on 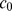 and 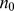, we have looked at the convergence and stability properties of these rescaling factors [12]. The relative change in the values of 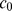 and 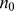 for all tags (in a particular year) was close to zero, suggesting that the rescaling factors converge quickly and are quite stable.

We have already shown in Tables 1 and 2 how 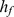 identifies top authors in their respective fields. To show how 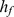 also allows to compare the impact of authors across disciplines, let us consider the example of two authors, S.H. Snyder in neurosciences and H. Garcia-Molina in computer science. Their impact cannot be compared based on the 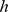-index as the two disciplines have different numbers of authors, publications, and citation patterns. Indeed, Snyder has 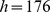 (global rank 6) while Garcia-Molina has 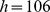 (global rank 60), suggesting that the former has a greater impact than the latter in absolute terms. However, when we compare the two based on the universal 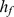-index, we find them in an effective tie for global rank 11 (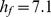). Indeed, both authors are equally successful (ranked first) in their respective fields.

**Figure 10:**
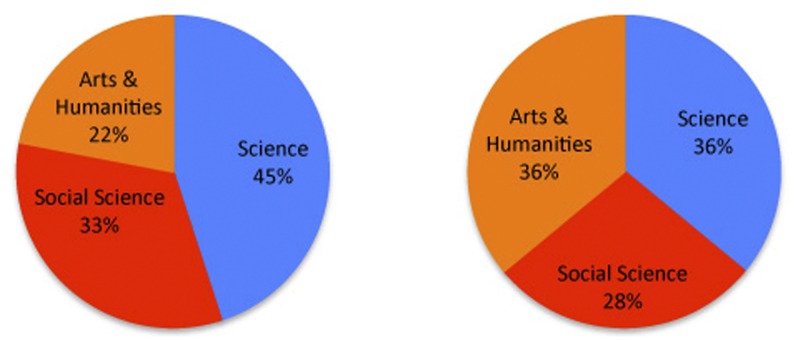
Distribution of JCR categories for top 100 authors based on 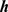
 **(left) and** 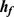 **(right) selected from a balanced sample of 3000 authors.** The 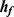-index leads to a more balanced representation of diverse fields.

For a quantitative evaluation of the universality of 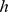 and 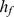 metrics, we follow the approach from Radicchi *et al.* to observe whether top authors from different areas are equally represented. To this end, let us compare the distribution of top authors based on the three JCR indices. We have approximately 20,000 reliable author-discipline annotations in *science* disciplines, 6,300 in the *social sciences*, and 1,750 in *arts & humanities*. Given such an unbalanced set, we sample 1,000 random authors from each set of disciplines. The authors in the sample are ranked by 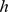 and 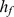 metrics, and the top 100 are selected based on each metric. This process is repeated 1,000 times. The resulting distributions of category tags for the top 100 authors are shown in Figure 10. The distribution based on 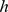 displays a clear bias toward science disciplines (45%), followed by social sciences (33%) and the least represented arts & humanities (22%). The 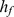 metric is not as biased, preserving a much better balance (36%, 28%, 36% respectively). This supports the universality claim, suggesting that the impact of authors in different disciplines can be compared in a more meaningful way using the 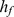 metric.

Another way to verify that 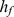 is a more universal citation impact metric than 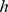 is to look at the distribution of impact values across disciplines. While we expect different authors within a discipline to have different impact, a universal metric should make different disciplines comparable. Let us therefore consider the average values of 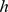 and 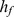 across the top 250 disciplines based on number of authors. A more universal measure should have a smaller variance across disciplines. However, the values of 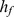 tend to be smaller than those of 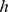, therefore to compare the variances let us define the normalized 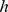 of discipline 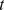 as 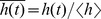, where 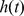 is the average 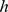 of authors in discipline 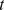 and 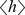 is the average across all disciplines. The normalized 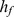, 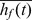, is defined analogously. The respective variances are 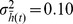 and 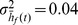. A Levene test of equality of variances reveals that the difference is very significant (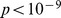). We conclude that 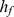 makes it more fair to compare impact in different disciplines. An illustration is presented in Table 3. The top disciplines based on average 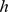 are dominated by life sciences, with a few exceptions such as theoretical physics. The life sciences tend to have more authors who publish a lot compared to other disciplines. Top tags based on average 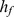 have greater diversity, ranging from biology to geosciences, materials science, and atmospheric sciences.

**Table 3:** Top disciplines based on 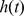 and 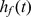 values, defined in the text as averages across all authors tagged with discipline 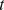.

	Discipline	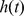	Discipline	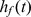
1	hematology	35	ophthalmology	1.93
2	obesity	34	geosciences, multidisciplinary	1.75
3	physics, theoretical	33	neuroimaging	1.72
4	gastroenterology & hepatology	32	materials science, multidisciplinary	1.71
5	immunology	31	clinical neurology	1.70
6	biostatistics	30	meteorology & atmospheric sciences	1.68
7	medicine	29	geochemistry & geophysics	1.63
8	nutrition & dietetics	29	radiology, nuclear medicine & medical imaging	1.62
9	medicine, research & experimental	29	pathology	1.60
10	neuroimaging	27	psychology, experimental	1.59

We considered disciplines with at least 20 authors (as of April 2012).

## Conclusions

### Summary

We introduced a Web Science approach to gather scholarly metadata. We presented Scholarometer, a social Web tool that leverages crowdsourced scholarly annotations with many potential applications, such as bibliographic data management, citation analysis, science mapping, and scientific trend tracking. We discussed a browser-based architecture and implementation for the Scholarometer tool, affording platform and source independence while complying with the usage policy of Google Scholar and coping with the noisy nature of the crowdsourced data. We outlined disambiguation algorithms to deal with the challenge of common author names, by incorporating a classifier into the query manager.

We found evidence that the crowdsourcing approach can yield a coherent emergent classification of scholarly output. The annotation and citation metadata that we collect is shared with the research community via an API and linked open data. By combining a visualization of disciplinary networks with lists of high-impact authors into an interactive application, the Scholarometer system can be a powerful resource to explore relevant scholars and disciplines. Interactive author networks can help one identify influential authors in one's discipline or in interdisciplinary or emerging areas.

We outlined several citation-based impact metrics that are computed by the Scholarometer tool, including the first implementation of the universal 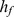-index. We also found that the statistics collected by our social tool make the 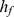 metric more appropriate compared to the original 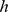-index for comparing the impact of authors across disciplinary boundaries.

### Future Work

Of course, as the crowdsourced database grows, our data for each discipline will become more representative and our measures more reliable.

Additional metrics can be implemented, for instance universal ones based on percentiles [36] and the successive 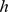-index for groups [37], which could be used to rank department-like units. We also plan to compute *temporal metrics*, i.e., to track an author's impact backward in time. This would allow to compare authors at the same stage in their career, even if they are not contemporary.

Studies of co-authorship patterns in conjunction with citation patterns might help further characterize the structure and evolution of disciplines. Moreover, by tracking the spikes in the popularity of disciplines, we plan to explore trends in scientific fields, in particular how disciplines emerge and die over time.
